# Modes of tetra(4-pyridyl)porphyrinatomanganese(III) ion intercalation inside natural clays

**DOI:** 10.1186/s13065-016-0153-4

**Published:** 2016-03-10

**Authors:** Ahed Zyoud, Waheed Jondi, Waseem Mansour, M. A. Majeed Khan, Hikmat S. Hilal

**Affiliations:** SSERL, Department of Chemistry, An-Najah National University, Nablus, West Bank Palestine; King Abdullah Institute for Nanotechnology, King Saud University, P.O. Box 2454, Riyadh, 114 51 Saudi Arabia

## Abstract

**Background:**

Metalloporphyrin ions, with planar shape, have been known to intercalate horizontally and diagonally between montmorillonite layers. Perpendicular intercalation inside montmorillonite has not been reported earlier. This work aims at achieving perpendicular intercalation inside montmorillonite in natural clays. Possible intercalation inside other forms of natural clay will also be investigated.

**Methods:**

Natural clays were purified and characterized. The naked clay powder was then refluxed with tetra(4-pyridyl)porphyrinatomanganese(III) ion (MnTPyP^+^) solution in methanol with continuous stirring for different times. Electronic absorption spectra, atomic absorption spectra, Fourier Transform infrared spectra, scanning electron microscopy and X-ray diffraction were all used in clay characterization and in intercalation study.

**Results:**

The natural clay involved different phases, namely montmorillonite, biotite, kaolinite, illite and traces of quartz. Montmorillonite clay allowed horizontal, diagonal and perpendicular intercalation of the metalloporphyrin ions. Biotite allowed only horizontal intercalation. The mode of intercalation was deduced by monitoring the clay inter-planar distance value change. Intercalation occurred inside both micro- and nano-size clay powders to different extents. The nano-powder (average size ~50 nm) showed uptake values up to 3.8 mg MnTPyP/g solid, whereas the micro-size powder (average size ~316 nm) exhibited lower uptake (2.4 mg MnTPyP/g solid). Non-expandable clay phases did not allow any intercalation. The intercalated MnTPyP^+^ ions showed promising future supported catalyst applications.

**Conclusions:**

Depending on their phase, natural clays hosted metalloporphyrin ions. Montmorillonite can allow all three possible intercalation geometries, horizontal, diagonal and for the first time perpendicular. Biotite allows horizontal intercalation only. Non-expandable clays allow no intercalation.

**Electronic supplementary material:**

The online version of this article (doi:10.1186/s13065-016-0153-4) contains supplementary material, which is available to authorized users.

**Graphical abstract:**
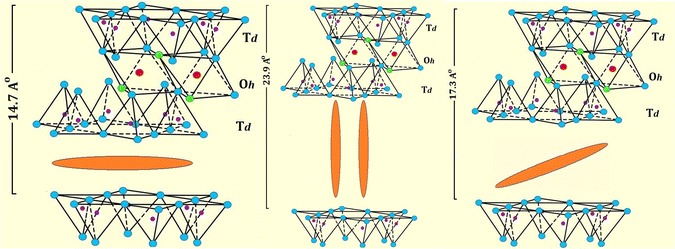
Metalloporphyrin complexes can be intercalated into montmorillonite in horizontal, perpendicular and diagonal geometries

## Background

Metalloporphyrins are a widely studied class of compounds [1–5]. With their planarity, aromaticity and stability, they have been used as thermal catalysts [6–12], photo-catalysts [13], and electro-catalysts [14, 15]. MnTPyP^+^ ions are useful homogeneous catalysts, but to facilitate their recovery they have been supported onto different types of insoluble solid materials. The ions were chemically anchored to solid material surfaces [9, 16]. They were also encapsulated inside clay and zeolite cavities [17–21].

Clays involve two-dimensional layers of SiO_4_ tetrahedra and/or AlO_4_ octahedra, with the general formula (Al,Si)_3_O_4_. The clay layers involve arrays of tetrahedral stack sided by octahedral stack (in Kaolinite). Alternatively, a stack of octahedral layer can be sided by two tetrahedral stacks (in Montmorillonite). The surfaces of each layer involve OH groups, which allow inter-planar H-bond attractions that hold the total structure intact [19]. Depending on the combinations of the silicon and aluminum ions, the clay frameworks carry net zero or negative charges. The negative charges are balanced by foreign cations such as Na^+^, Mg^2+^, or others.

Clays are potentially useful to support MnTPyP^+^ ions on the surface by adsorption [22] or by genuine chemical anchoring [23, 24]. The MnTPYP^+^ ions can also penetrate between the clay layers and replace the foreign cations by ionic exchange [25]. This would be encouraged by the planar nature of MnTPyP^+^. Fujimura et al. reported that cationic metalloporphyrins were intercalated from water/ethanol solvent into clay membranes as separate ions without aggregation inside [26]. The planar ions were horizontally placed between the clay layers. Ma et al. reported that anionic metalloporphyrins were intercalated inside layered double hydroxides in a perpendicular orientation [27]. Metalloporphyrins intercalated inside layered double hydroxides have been witnessed earlier [20, 28]. Tubular halloyasites have also been described for metalloporphyrin intercalation purposes [29]. Constantino et al. reported the intercalation of metalloporphirins inside different types of inorganic solids, namely smectite clays, layered double hydroxides and layered niobates in their separate phases [19]. They showed that intercalation inside clays occurred horizontally, while in layered double hydroxides intercalation occurred perpendicularly. Niobate exhibited diagonal intercalation. In another more recent study, tetra(4-pyridyl)porphyrinato iron(III) ions were intercalated inside montmorillonite horizontally and diagonally [30] but not perpendicularly.

To our knowledge, perpendicular intercalation of metalloporphyrins inside clays has not been reported so far. Despite that, there is no reason to rule out perpendicular intercalation of metalloprphyrine ions inside montmorillonite. The main goal of this work is to investigate possible intercalation of MnTPyP^+^ ions inside natural clays, in a perpendicular manner, for the first time. With perpendicular intercalation, the clay layers will become highly open, and the MnTPyP^+^ ion catalysts will be more accessible to reactants. The resulting supported catalysts would thus have enhanced characteristics compared to their homogeneous counterparts.

Ceramics made from natural clays, taken from northern areas of the Palestinian Territories, were chosen to host MnTPyP^+^ ions. Such ceramic materials are abundant, low-cost and non-hazardous. They are commonly used in mosaics, drinking water potteries and other traditional hand-crafts. The naked clays will first be characterized to know their phases and crystal structures. Intercalation study will then be performed on such clays. Both micro- and nano-size powders will be examined. The nano-powder is anticipated to exhibit higher MnTPyP^+^ uptake capacity than the micro-powder [31].

## Experimental

Chemicals and solvents were purchased from Aldrich Co and Riedel-de Haen. Pre-calcinated ceramics (400 °C, 24 h), made from natural clays collected from Jenin area of the North West Bank, Palestinian Territories, were treated and cleaned as described below.

A Shimadzu UV-1601 spectrophotometer from LaboMed, Inc. was used for electronic absorption spectral analysis. Solid state vibrational spectra were measured on a Thermo Fisher-ASB1200315-Nicolet 5 FT-IR Spectrometer. Specific surface area values were measured by adsorption of acetic acid from organic solvent, as described earlier [32].

AAS data were measured on an ICE 3000 AA spectrometer from Thermo Scientific. FE-SEM micrographs were measured on a Jeol Model JSM-6700 F microscope in the laboratories of King Sa’ud University, Riyadh, Saudi Arabia. XRD patterns were measured on a Philips XRD X’PERT PRO diffractometer with CuK α (λ_1.5418A_) as a source, in the laboratories of the King Sa’ud University, Riyadh, Saudi Arabia.

### Cleaning the natural clay

Pre-calcinated clay solid (50.0 g) was ground and sieved. The 30–100 mesh was taken and soaked in nitric acid (200 ml, 50:50 v/v) for 24 h to remove metal ion impurities. The resulting white solid was then separated by suction, and carefully washed with deionized water many times until neutral. The clay was dried in an oven at 120 °C for 2 h and stored in a desiccator. This clay is termed Naked Clay here. Different ions existed inside the pre-calcinated natural clay and were analyzed in the nitric acid solution, by atomic absorption spectra, including Ca^2+^ (1.0 %), Fe^2+^/Fe^3+^ (~2.0 %), Mg^2+^ (0.5 %) and others in trace amounts.

### Preparation of tetra(4-pyridyl)porphyrinato-manganese(III) *[Mn*^*III*^*(Tpyp)]*^+^*(SO*_*4*_*)*_*1/2*_

Tetra(4-pyridyl)porphyrinato-manganese(III) sulfate [Mn^III^(TPyP)]^+^ was prepared in batch samples as described in literature [24]. N,N-dimethylformamide (DMF) (180 ml) was magnetically stirred with 5,10,15,20-tetra(4-pyridyl)21H,23H-porphin (490 mg, 0.792 mmol) and excess MnSO_4_·H_2_O (0.900 g, 5.45 mmol) in a round-bottomed flask. The mixture was then refluxed under air stream for 10 h. The UV-visible spectrum measured in DMF confirmed the product with its characteristic bands at 569, 512 and 463 nm (Soret). The solvent was removed under suction while allowing a stream of air to complete oxidation of manganese. Column chromatography, with neutral alumina (Bio-Red AGF, 100–200 mesh) as a stationary phase, was used to separate the product in pure form. Elution was performed with a mixture of methanol/chloroform (15:85 v/v). The eluent which contained Mn^III^(TPyP) ions was taken and dried under reduced pressure at room temperature. The final batch product (480.00 mg, 0.669 mmol) was collected and stored in solid form.

### Intercalation of MnTPyP^+^ ions inside clay particles

A solution of MnTPyP^+^ (4.17 × 10^−3^ M) in methanol was prepared by dissolving [Mn^ш^(TPyP)](SO_4_)_1/2_ (0.300 g, 0.417 mmol) in methanol (100.0). The concentration was further confirmed by AAS. The solution was mixed with 15.0 g of pre-cleaned naked clay, and the mixture was refluxed with vigorous magnetic stirring for 30 h under dry atmosphere, using a CaCl_2_ drying tube. The electronic absorption spectra for the reaction mixture were measured for aliquots (1.0 ml) syringed out from the mixture. The solid material was then carefully filtered and rinsed with methanol to remove any remaining free or surface-adsorbed MnTPyP^+^ ions. The remaining (non-intercalated) metalloporphyrin concentration was calculated by AAS. The resulting solid was then dried and named MnTPyP@Nano-Clay.

To intercalate the MnTPyP^+^ into micro-scale clay, the same technique was followed using reflux with vigorous stirring for only 6 h. The resulting solid was termed MnTPyP@Micro-Clay. The solid was pale yellowish, compared to MnTPyP@Nano-Clay solid which showed more intense reddish color. The change in the Naked Clay white color is an indication of metalloperphyrin presence. In each case the intercalated amount of MnTPyP^+^ was calculated using AAS. Calibration curves were constructed for this purpose.

Control experiments were performed. In one experiment, Naked Clay was refluxed in methanolic solution of MnTPyP^+^ ions with no magnetic stirring. In another experiment, Naked Clay was refluxed in methanol with magnetic stirring in the absence of MnTPyP^+^ ions. Clay grinding occurred in the second control experiment, which indicates that grinding is due to magnetic stirring. Stirring under reflux for longer than 30 h produced ultra-fine powders which were difficult to handle in the dried form.

## Results and discussion

Refluxing the Naked Clay powder with methanolic solution of MnTPyP^+^ ions, with magnetic stirring, caused grinding of the clay powder into micro- or nano-scale powders. Intercalation was then confirmed by different methods. The three solids, Naked Clay, MnTPyP@Nano-Clay and MnTPyP@Micro-Clay have been characterized by different techniques.

### FT-IR spectral analysis

**Fig. 1:**
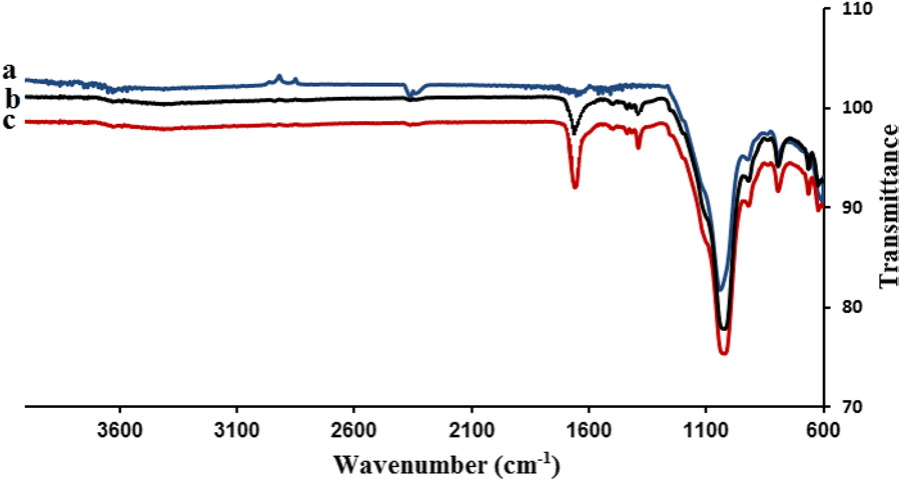
Solid state FT-IR spectra measured for (*a*) natural clay powder, (*b*) MnTPyP@Micro-Clay and (*c*) MnTPyP@Nano-Clay

The presence of the MnTPyP^+^ ions inside the composite material was confirmed by FT-IR spectra. Fig. (1b, c) show that each of MnTPyP@Micro-Clay and MnTPyP@Nano-Clay has two new major bands at 1650 and 1385 cm^−1^. The two bands correspond to the two bands (1656 and 1388 cm^−1^) observed for the homogeneous MnTPyP^+^ ions dissolved in methanol solvent here. The two bands also resemble the ones at 1600 and 1380 cm^−1^ reported for other polysiloxane supported tetra(4-pyridyl)porphyrinato manganese(III) complexes [33, 34].

A closer look at the spectra shows other evidence in favor of intercalation. The band at 1026 cm^−1^ observed for the Naked Clay powder in Fig. 1a was shifted to 1010 cm^−1^ after introduction of the MnTPyP^+^ ions. This indicates that the bonds at the clay layer surfaces were affected by intercalation of the MnTPyP^+^ ions in between.

The FT-IR spectra thus confirm intercalation of MnTPyP^+^ ions inside the clay. Presence of additional MnTPyP^+^ ions adsorbed at the outer surface of the clay cannot be ruled out, despite the careful rinsing of the resulting composite after reflux.

### Electronic absorption spectral analysis

**Fig. 2:**
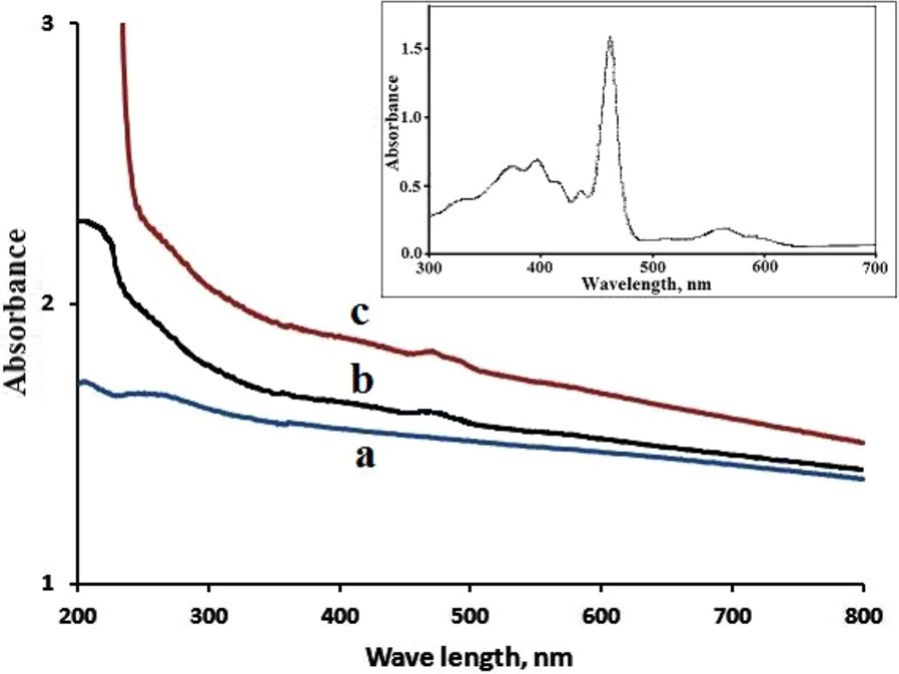
Electronic absorption spectra measured for aqueous suspensions of (*a*) Naked Clay, (*b*) MnTPyP@Micro-Clay and (*c*) MnTPyP@Nano-Clay powders. The* inset* shows the spectrum for MnTPyP^+^ ions dissolved in DMF

Electron absorption spectra, Fig. 2, also confirmed the presence of the MnTPyP^+^ ions inside the MnTPyP@Micro-Clay and MnTPyP@Nano-Clay. Fig. 2a shows the spectrum for the Naked Clay in the absence of MnTPyP^+^ ions. With its white color, the clay is expected to show no bands in the visible region. The two bands at 470 and 490 nm in Fig. 2c correspond to the Soret band for the in solution MnTPyP^+^ ions shown in the inset. In the free or solution forms of MnTPyP^+^, the Soret band typically occurs at about 462 nm, depending on type of solvent [9, 33, 35]. The composite MnTPyP@Micro-Clay, Fig. 2b, shows the same two bands at 470 and 490 nm for MnTPyP^+^ ions, with weaker intensities due to lower concentration of intercalated ions. The difference in MnTPyP^+^ bands in Fig. 2b, c is consistent with the color difference discussed in “Intercalation of MnTPyP^+^ ions inside clay particles” section above. The measured concentrations discussed below also confirm these results.

Both Fig. 2b, c involve a red shift in Soret band for the MnTPyP^+^ ions. Such a red shift confirms intercalation. The presence of two bands indicates different types of intercalated MnTPyP^+^, as will be further discussed.

### AAS analysis

AAS was used to calculate the exact amount of [Mn^III^(Tpyp)]^+^(SO_4_)_1/2_ intercalated inside different clay powders. The amounts of excess solution MnTPyP^+^ and those eluted from the surface of the clay were grouped together and subtracted from the initial nominal amount originally used. The uptake of MnTPyP^+^ inside MnTPyP@Micro-Clay was 2.37 mg/g clay (3.12 × 10^−3^ mmol/g). Higher MnTPyP^+^ uptake occurred in the MnTPyP@Nano-Clay with 3.78 mg/g (5.25 × 10^−3^ mmol/g). This is expected as the nano-scale particles have higher relative surface area, vide infra. With smaller clay particle sizes, the MnTPyP^+^ ions also have shorter path length to travel inside. The difference in color intensity between the MnTPyP@Nano-Clay and MnTPyP@Micro-Clay further confirms these results.

Refluxing the Naked Clay with methanolic solution of MnTPyP^+^ ions for prolonged times may still yield higher uptake. Saturation uptake value may increase with further grinding in the clay particles. However, for practical handling purposes and to avoid further grinding of the clay into smaller sizes, the reflux/stirring time was not extended for longer than 30 h.

Ion exchange study was performed on the Naked Clay powder in its H-form as described by common procedures described earlier using aqueous Na^+^ ion solutions [36]. The exchange capacity was ~0.8 mmol/g (18.4 mg Na^+^/g). The value is lower than other literature values [30] because the clay here involves layered montmorillonite and biotite in addition to other non-layered phases, as described below. Moreover, the MnTPyP^+^ ion uptake values in both nano- and micro-scale clay powders are lower than the Na^+^ cation exchange capacity of the clay, as discussed above. This is not unexpected, as the MnTPyP^+^ ions may not be able to reach all negative sites inside the clay. Similar behavior has been reported earlier [30].

### Specific surface area

The specific surface area for each of the three solids was measured by acetic acid adsorption from organic solvents [32]. From literature, the specific surface area for clay is not easy to measure accurately [37]. The values may have wide variations, depending on type of the clay, the particle size and the technique used. The approximate values measured here were 90, 130 and 200 m^2^/g for Naked Clay, MnTPyP@Micro-Clay and MnTPyP@Nano-Clay powders, respectively. Despite being only rough estimates, the values still give indication that the MnTPyP@Nano-Clay has highest specific surface area among the series, which explains why it exhibited higher MnTPyP^+^ ion uptake, as discussed above.

### SEM micrographs

**Fig. 3:**
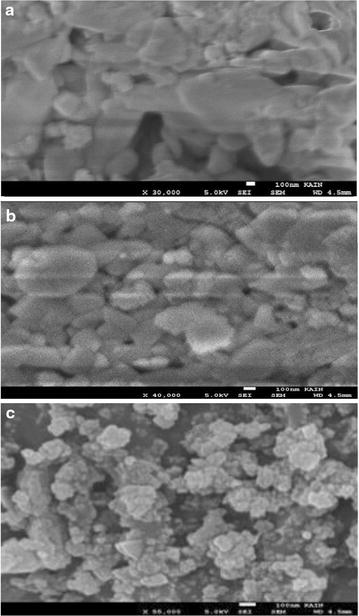
SEM micrographs measured for (**a**) Naked Clay, (**b**) MnTPyP@Micro-Clay and (**c**) MnTPyP@Nano-Clay

The naked and intercalated clay surfaces were studied with FE-SEM, Fig. 3. SEM micrographs were used to measure the sizes of the three types of clay particles. The Naked Clay showed particle sizes in the range 200–1000 nm with an average value of 625 nm. The MnTPyP@Micro-Clay particles showed a size range of 100–600 nm, with an average radius of 316 nm. The MnTPyP@Nano-Clay particles showed sizes in the range 10–140 nm with an average radius of 50 nm.

The SEM images clearly confirm grinding of the Naked Clay particles (under 6 h magnetic stirring and reflux) into smaller particles of MnTPyP@Micro-Clay. Further grinding into nano-scale particles has been achieved by longer stirring (30 h) under reflux conditions. Clay particle grinding during reflux made it not possible to observe size expansion due to intercalation with SEM.

### XRD patterns

**Fig. 4:**
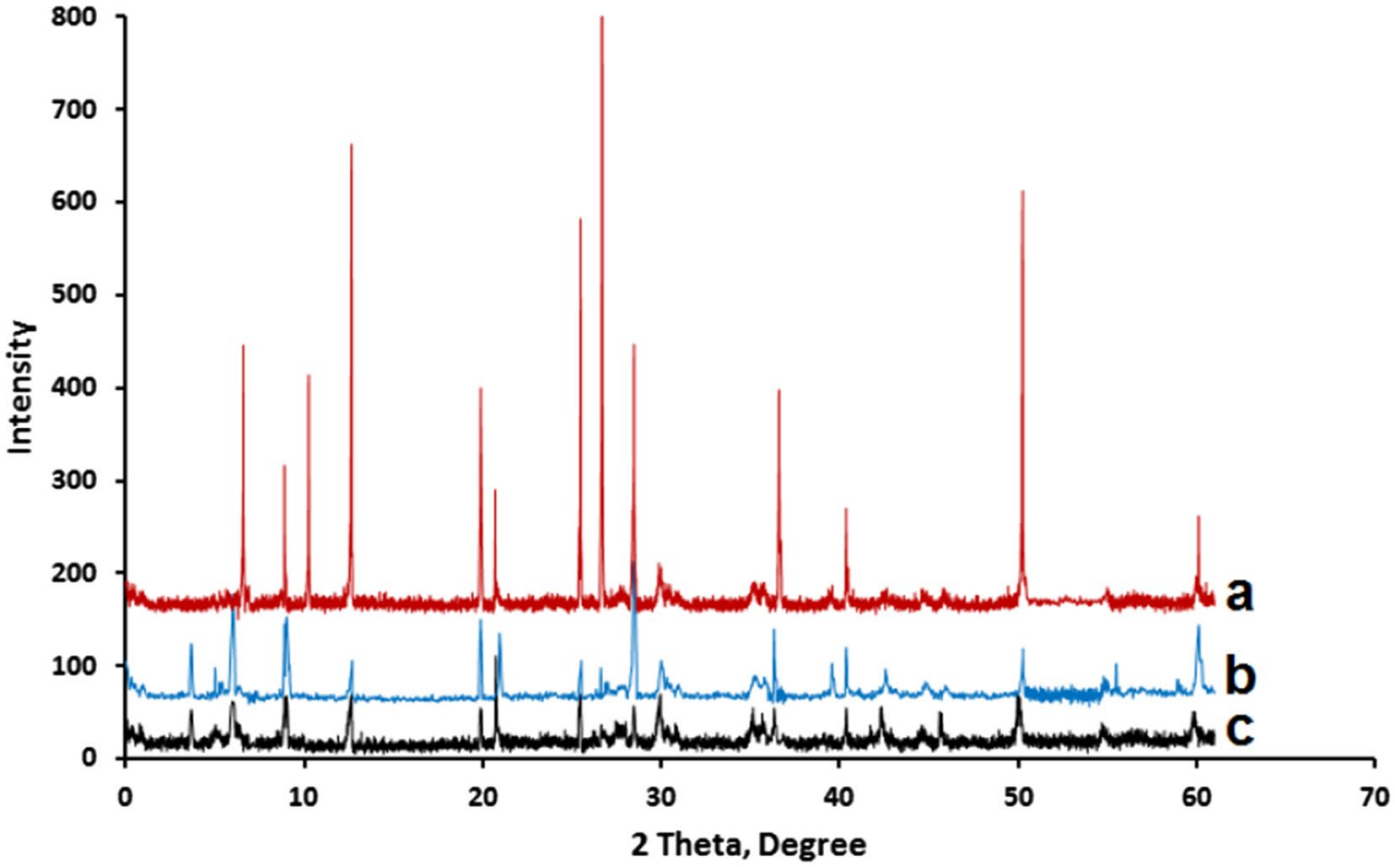
XRD patterns measured for (*a*) Naked Clay, (*b*) MnTPyP@Micro-Clay and (*c*) MnTPyP@Nano-Clay

Figure 4 shows the XRD patterns measured for Naked Clay, MnTPyP@Micro-Clay and MnTPyP@Nano-Clay powders. The XRD patterns were mainly used to confirm the intercalation of MnTPyP^+^ ions inside the clay particles. The intercalation orientation was also studied by the patterns.

Comparison of the XRD patterns in the Figure with earlier reports [38] shows that the Naked Clay contains Kaolinite (with 2θ = 12.65°, 25.48°, 40.36° and 55°). Signals for quartz appear (at 2θ 20.70°, 26.69°, 50.26° and 60.11°). Illite also exists inside the Naked Clay (with 2θ 8.89° and 36.60°). Montmorillonite presence is evident in the Naked Clay (with 2Ɵ 6.58, 19.88 and 28.47 degree). Furthermore, the Naked Clay involves biotite (with 2θ 10.25°). The Montmorillonite and Kaolinite are the dominant phases, while the others are minor phases. All such phases are confirmed by comparing Fig. 4a with literature.

Figure 4a shows relatively sharp and high signals for Naked Clay, which means that its particles are relatively more crystalline than the other two powders. The signals became shorter and broader due to grinding by stirring under reflux for 6.0 h as shown in Fig. 4b. After longer treatment (30.0 h) the MnTPyP@Nano-Clay signals were further broadened and shortened. The signals are typical for nano-scale particles with lower crystallinity. The XRD patterns are consistent with the SEM micrographs discussed above.

**Fig. 5:**
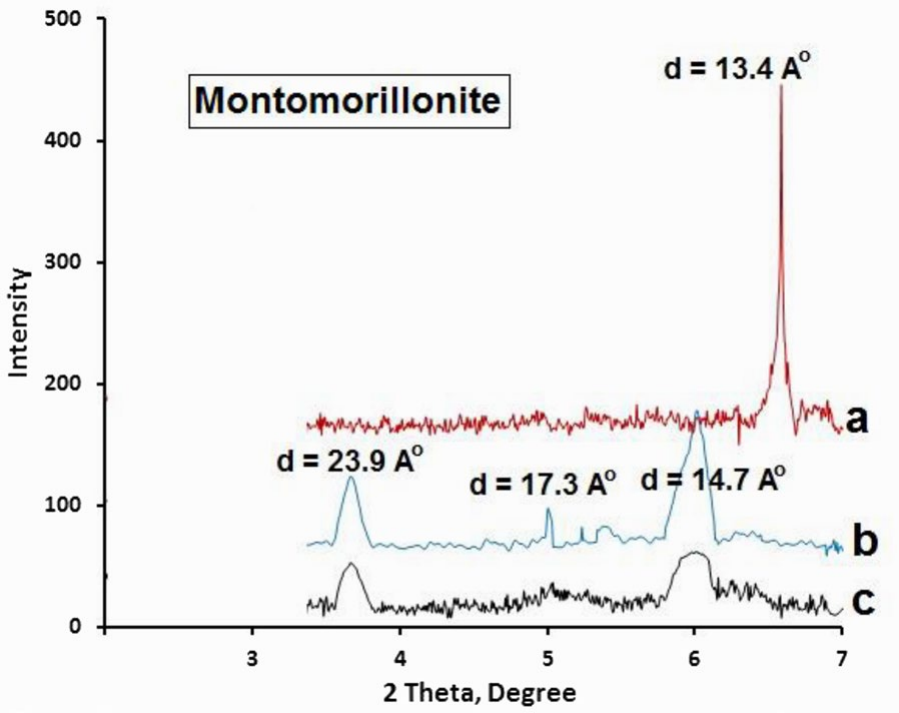
Summary of XRD shifting in 2θ values and corresponding D values for montmorillonite phase. (*a*) Naked Clay, (*b*) MnTPyP@Micro-Clay and (*c*) MnTPyP@Nano-Clay

The XRD patterns gave insight on orientation of MnTPyP^+^ ions intercalated inside the expandable montmorillonite phase. Figure 4a–c show shift in value of 2θ from (6.58 degree, for Naked montmorillonite) to three new values (6.00, 5.00 and 3.66 degree), after refluxing with MnTPyP^+^ for both MnTPyP@Micro-Clay and MnTPyP@Nano-Clay. This indicates interlayer distance expansion as a result of metalloporphyrin penetration between clay layers. The three different shift values indicate three intercalation orientations: horizontal, perpendicular and diagonal. The shifts in montmorillonite signal are re-summarized in Fig. 5.

Unlike earlier reports discussed above, the XRD patterns here indicate that the montmorillonite phase exhibited three different types of intercalation. Based on Braggs’ law, and taking into consideration λ = 1.54 Å, n = 1, and 2θ = 6.58 degrees, the original distance between tops of two adjacent layers (**d)** equals 13.4 Å. This value is consistent with earlier reports [39–41].

**Fig. 6:**
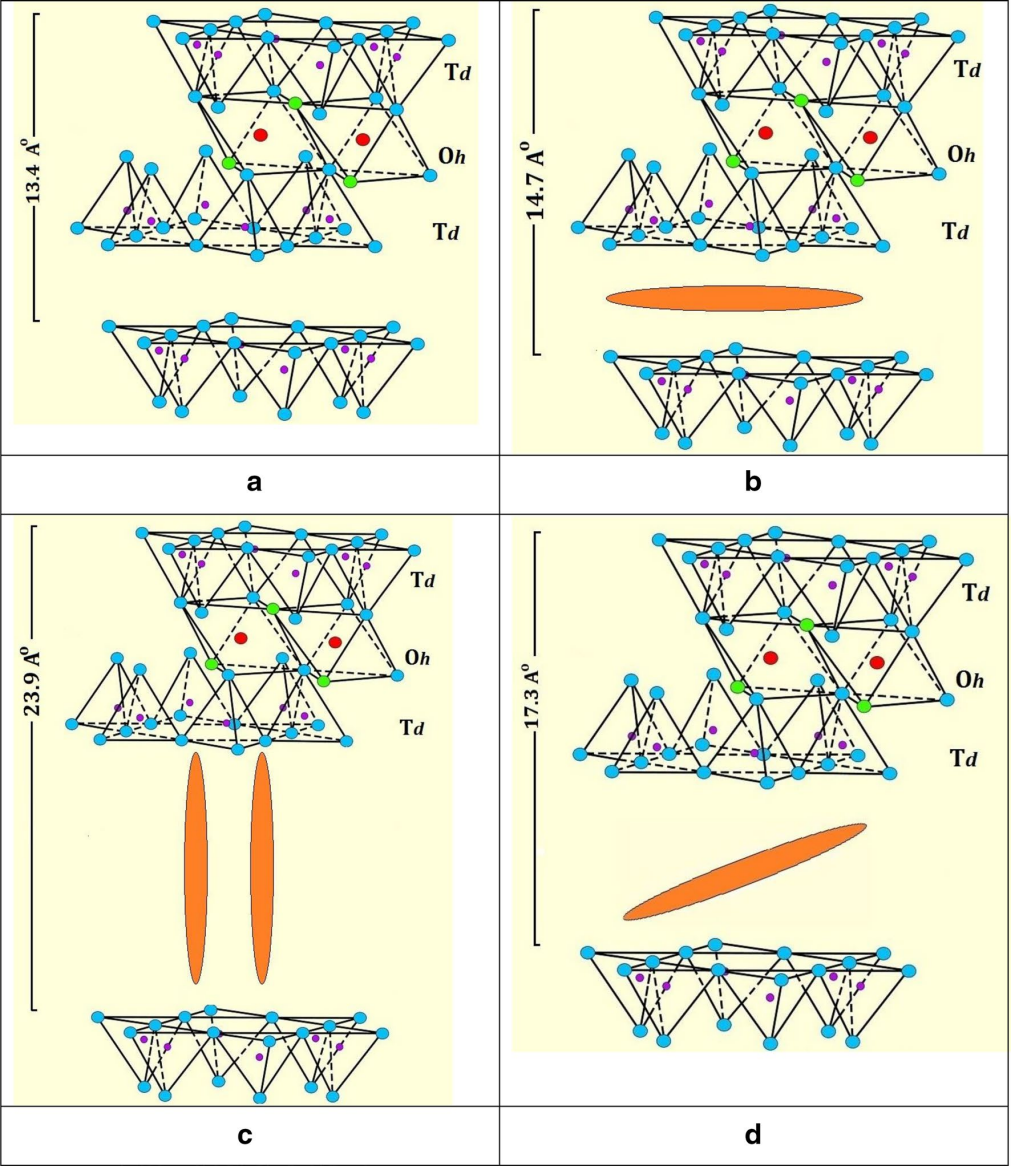
Schematic showing different intercalation orientations of MnTPyP^+^ ion between two* adjacent* montmorillonite layers. (**a**) Naked Clay, (**b**) Horizontal orientation, (**c**) Perpendicular orientation and (**d**) Diagonal orientation. The scheme is reproduced from literature [43]

The shifting from 6.58° to 6.00° indicates that **d** increased from 13.4 Fig. 6a to 14.7 Å Fig. 6b. The net spacing expanded by about 1.3 Å. The MnTPyP ion has a thickness of about 1.09 Å. The results indicate that a monolayer of metalloporphyrin ions is sandwiched horizontally between two adjacent layers as shown in Fig. 6b. The logic is based on the concept of size matching effect reported earlier [42].

The shifting from 6.58° to 5.00° means that **d** increased from 13.4 to 17.3 Å, with net expansion of 3.9 Å. This indicates that the metalloporphyrin ions intercalated between two adjacent layers in a diagonal manner, as shown in Fig. 6d. By this way, the metalloporphyrin intercalation caused expansion by 3.9 Å.

Shifting the signal from 6.58° to 3.66° indicates increase in the distance between the tops of two adjacent layers (**d**) from 13.4 to 23.9 Å by intercalation, as shown in Fig. 6c with net expansion of ~10.5 Å. This means that the metalloporphyrin ions are perpendicularly inserted between two adjacent layers. Similar logic has been used by Constantino et al. for other metalloporphyrin systems perpendicularly intercalated in non-clay solids [19] as discussed above. Such expansion of space between adjacent layers is expected to facilitate entrance of different reactants inside the clay and consequently speed up catalytic organic reactions therein. The resulting supported catalyst will thus have an added value, as reported earlier [17].

Horizontal and perpendicular intercalations of metalloporphyrins inside different materials are known [42, 44, 45]. Diagonal orientations have also been reported inside niobite Nb_3_O_8_^−^ [19]. To our knowledge, perpendicular intercalations have not been described in montmorillonite in earlier reports. This work manifests perpendicular intercalation inside montmorillonite for the first time.

**Fig. 7:**
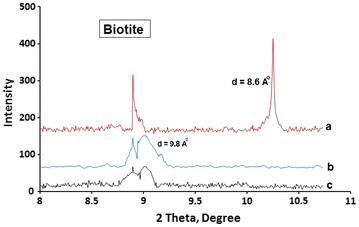
Summary of XRD shifting in 2ϴ values and corresponding D values for biotite phase. (*a*) Naked Clay, (*b*) MnTPyP@Micro-Clay and (*c*) MnTPyP@Nano-Clay

With its planar structure, biotite is expected to host MnTPyP^+^ ions by intercalation. RXD pattern, Fig. 4a–c, shows shifting in 2θ from 10.25° to 9.01°. The results are re-summarized in Fig. 7 a,b and c. The **d** value expanded from 8.6 to 9.8 Å, with only 1.2 Å expansion. This indicates that MnTPyP^+^ ions intercalated into biotite layers only horizontally. Both micro- and nano-clays underwent intercalation as shown in Fig. 7.

Kaolinite and Illite phases did not exhibit intercalation with MnTPyP^+^ ions. This is due to their well-known relatively small interlayer distances, as they belong to the non-expandable clays. Due to this reason they are used in ceramic industry, because they do not absorb water molecules. Quartz does not have separate layer structure, and consequently does not allow MnTPyP^+^ intercalation.

The catalytic activity of the intercalated MnTPyP^+^ ions, described here, in olefin hydrosilylation reaction has been reported recently. Reactions were conducted using Naked Clay, MnTPyP@Nano-Cla and homogeneous MnTPyP^+^ ions. While the Naked Clay showed no catalytic activity, the intercalated catalysts showed higher activity and selectivity than the homogeneous metalloporphyrin ion. The MnTPyP@Nano-Clay catalyst exhibited a turnover frequency (up to 1200 min^−1^) and exceptionally high selectivity to produce terminal hydrosilylation reaction products. Soundly high activity on recovery and reuse for third time was also observed for the MnTPyP@Nano-Clay system. An explanation for these behaviors has been discussed based on a proposed mechanism [46]. With its expanded layer structure, the clay allowed the reactant molecules, tri(ethoxy)silane and 1-octene, to reach the supported MnTPyP^+^ catalyst sites. Moreover, the cavities inside the clay support exhibited solvent like behavior and increased the catalyst efficiency. Unlike the homogeneous catalyst system, the supported catalyst showed high selectivity to produce terminal hydrosilylation product only based on steric effect.

Work is under way to investigate catalytic activity of clay supported metalloporphyrins in other types of reactions. Using single pure forms of clays, such as montmorillonite and biotite, as supports for different metallporphyrin ion catalysts is also underway (Additional file 1).

## Conclusions

Pre-calcinated powder, made of naturally occurring clay from Northern areas of the West Bank, Palestinian Territories, involves five different phases. When refluxed under magnetic stirring, the Naked Clay particles were ground into micro- or nano-scale particles. Metalloporphyrin was intercalated into layers of montmorillonite and biotite phases of natural clay ground to micro- and to nano-scales. Montmorillonite allowed intercalation of the metalloporphyrin ions in perpendicular, horizontal and diagonal fashions. The biotite allowed horizontal intercalation only. Non expandable forms, illite and kaolinite, did not allow any intercalation. AAS and XRD confirmed the intercalation results. Preliminary studies indicate that the new MnTPyP@Clay systems showed relatively high catalytic activity and selectivity in olefin hydrosilylation reactions.

## Additional file

10.1186/s13065-016-0153-4
