# Supplementary material for: Modes of tetra(4-pyridyl)porphyrinatomanganese(III) ion intercalation inside natural clays
Source: Chem Cent J. 2016 Mar 10;10:12. doi: 10.1186/s13065-016-0153-4 (PMC4787189; doi:10.1186/s13065-016-0153-4)
Supplement: Supplementary file 1 — 10.1186/s13065-016-0153-4 [file 13065_2016_153_MOESM1_ESM.doc]

Highlights:

- Perpendicular intercalation of metalloporphyrin ions occurred inside natural montmorillonite.
- Other orientations (horizontal and diagonal) were also witnessed.
- In biotite only horizontal intercalation occurred.
- Intercalation occurred in nano-clay particles more profoundly than in the micro-particles.
- The prepared composite materials showed promising high catalyst activity
